# Dietary polyphenols as potential nutraceuticals in management of diabetes: a review

**DOI:** 10.1186/2251-6581-12-43

**Published:** 2013-08-13

**Authors:** Zahra Bahadoran, Parvin Mirmiran, Fereidoun Azizi

**Affiliations:** 1 Nutrition and Endocrine Research Center, and Obesity Research Center, Research Institute for Endocrine Sciences, Shahid Beheshti University of Medical Sciences, No 24 Parvaneh St, Yemen St, Chamran Exp, 19395-4763 Tehran, Iran; 2 Department of Clinical Nutrition and Dietetics, Faculty of Nutrition Sciences and Food Technology, National Nutrition and Food Technology Research Institute, Shahid Beheshti University of Medical Sciences, No 46 Arghavan-e-gharbi St, Farahzadi Blv, Shahrak-e-Ghods, 19395-4741 Tehran, Iran; 3 Endocrine Research Center, Research Institute for Endocrine Sciences, Shahid Beheshti University of Medical Sciences, No 24 Parvaneh St, Yemen St, Chamran Exp, 19395-4763 Tehran, Iran

**Keywords:** Polyphenols, Phenolic acids, Flavonoids, Stilbenes, Lignans, Type 2 diabetes, Diabetes complications

## Abstract

In recent years, there is growing evidence that plant-foods polyphenols, due to their biological properties, may be unique nutraceuticals and supplementary treatments for various aspects of type 2 diabetes mellitus. In this article we have reviewed the potential efficacies of polyphenols, including phenolic acids, flavonoids, stilbenes, lignans and polymeric lignans, on metabolic disorders and complications induced by diabetes. Based on several *in vitro*, animal models and some human studies, dietary plant polyphenols and polyphenol-rich products modulate carbohydrate and lipid metabolism, attenuate hyperglycemia, dyslipidemia and insulin resistance, improve adipose tissue metabolism, and alleviate oxidative stress and stress-sensitive signaling pathways and inflammatory processes. Polyphenolic compounds can also prevent the development of long-term diabetes complications including cardiovascular disease, neuropathy, nephropathy and retinopathy. Further investigations as human clinical studies are needed to obtain the optimum dose and duration of supplementation with polyphenolic compounds in diabetic patients.

**Electronic supplementary material:**

The online version of this article (doi:10.1186/2251-6581-12-43) contains supplementary material, which is available to authorized users.

## Introduction

Type 2 diabetes is a complex metabolic disorder associated with developing insulin resistance, impaired insulin signaling and β-cell dysfunction, abnormal glucose and lipid metabolism, sub-clinical inflammation and increased oxidative stress; these metabolic disorders lead to long-term pathogenic conditions including micro- and macro-vascular complications, neuropathy, retinopathy, nephropathy, and a consequent decrease in quality of life and an increase in the rate of mortality [1–3]. Among the multiple risk factors underling the incidence and progression of type 2 diabetes, diet is the main modifiable factor. An increasing number of epidemiological investigations show that diet rich in foods with high-content of phytochemicals, high total antioxidant capacity and polyphenolic compounds may be related to lower risk of diabetes and predisposing factors [4–9].

Based on the current understanding of pathophysiology of insulin resistance and type 2 diabetes, multiple pharmacological and non-pharmacological interventions have been developed with the aim of improving glycemic control and prevention of diabetes complications; in this area, recently the use of functional foods and their bioactive components have been considered as a new approach in the prevention and management of diabetes and its complications [10–13].

Due to their biological properties, polyphenols may be appropriate nutraceuticals and supplementary treatments for various aspects of diabetes mellitus. Our aim here is to review the current evidences in relation to several potential efficacies of these bioactive components on type 2 diabetes mellitus and related metabolic disorders. We obtained relevant articles, including *in vitro*, animal models and human studies with appropriate design, as well as review articles with good quality, published between 1990 to 2013, through searches of the Medline and PubMed databases. We first describe a brief introduction on dietary polyphenols, food sources and properties, and then summarize the evidence from *in vitro,* animal models and clinical studies.

### Dietary polyphenols: food sources, bioavailability and metabolism

Polyphenols are natural phytochemical compounds in plant-based foods, such as fruits, vegetables, whole grains, cereal, legumes, tea, coffee, wine and cocoa; more than 8000 polyphenolic compounds, including phenolic acids, flavonoids, stilbenes, lignans and polymeric lignans have been identified in whole plant foods [5]. These compounds are secondary metabolites of the plants that act as a defense against ultraviolet radiation, oxidants and pathogens [14].

Polyphenols may be classified into several categories based on the number of phenol rings and structural elements that bind these rings to one another [15]. Phenolic acids are approximately a third of the polyphenolic compounds in the diet and include two main classes hydroxybenzoic acid derivatives (protocatechuic acid, gallic acid, *p*-hydroxybenzoic acid) and hydroxycinnamic acid derivatives (caffeic acid, chlorogenic acid, coumaric acid, Ferulic acid, sinapic acid); berry fruits, kiwi, cherry, apple, pear, chicory and coffee are the foods with high content of these phenolic acids [16]. Flavonoids are the most abundant polyphenols in the human diet and more than 4000 types of these compounds have been identified. There are six subclasses of flavonoids including anthocyanins, flavonols, flavanols, flavanones, flavones and isoflavones; anthocyanins (cyanidin, pelargonidin, delphinidin, malvidin) are found in the berries family, red wine, red cabbage, cherry, black grape and strawberry. Flavonols, including quercetin, kampferol and myricetin, have been mainly detected in onion, curly kale, leeks, broccoli and blueberries. Isoflavones are other most important dietary flavonoids that include daidzein, genistein and glycitein; soybeans and soy products are the richest sources of these estrogen-like structure compounds. Lignans, diphenoilc components with phytoesterogenic activity, have been found in high concentrations in linseed and other grains and cereals. Stilbenes occur in the human diet in low quantities; resveratrol, one of the well studied compounds of these groups, is largely detected in grapes and red wine [5, 16, 17].

It is estimated that dietary intake of polyphenols is approximately 1 g/day [18, 19]. Bioavailability of these bioactive components is dependent on food preparation processes, gastrointestinal digestion, absorption and metabolism [20]. During the absorption pathway, dietary polyphenols must be hydrolyzed by the intestinal enzymes or colonic microflora, and then be conjugated in the intestinal cells and later in the liver by methylation, sulfation or glucuronidation [21]. Polyphenols consequently reach and accumulate in the target tissue and induce biological properties; the polyphenol derivates mainly excrete through bile and urine. Several studies showed rapid absorption of the polyphenolic compounds, such as procyanidins, quercetin and flavanols into plasma, with plasma concentrations peaking at 2 or 3 hours after ingestion [16].

Several biological activities and beneficial properties have been documented for dietary polyphenols, and some of the more well known ones include antioxidant, anti-allergic, anti-inflammatory, anti-viral and anti-microbial, anti-proliferative, anti-mutagenic, anti-carcinogenic, free radical scavenging, regulation of cell cycle arrest, apoptosis, and induction of antioxidant enzymes; more interestingly, dietary polyphenols could modulate some important cell signaling pathways such as nuclear factor kappa-B (NF-κB), activator protein-1 DNA binding (AP-1), extracellular signal-regulated protein kinase (ERK), phosphoinositide 3 (PI3) kinase/protein kinase B (Akt), mitogen-activated protein kinases (MAPK), and nuclear factor erythroid 2 related factor 2 (Nrf2) [22].

### Anti-hyperglycemic effects of polyphenols with regard to carbohydrate metabolism, β-cell function and insulin resistance

Impaired carbohydrate metabolism and developing insulin resistance is the main metabolic disorder in non-insulin dependent diabetes mellitus leading to hyperglycemia. Altered digestion and absorption of dietary carbohydrate, depletion of glycogen storage, increased gluconeogenesis and over output hepatic glucose, β-cell dysfunction, insulin resistance of peripheral tissue and defect in insulin signaling pathways are more important causes of hyperglycemia [23]. Although the use of oral anti-diabetic drugs including α-glucosidase inhibitors, biguanides, meglitinides, sulfonylureas, thiazolidindiones or insulin therapies are common clinical options in management of type 2 diabetes and hyperglycemia, but traditionally used natural agents have been considered for a long period of time. Among the known natural bioactive components and phytochemicals, recently polyphenols are very popular because of their anti-hyperglycemic effects, safety and non side-effects. Potential efficacy of polyphenols on carbohydrate metabolism and glucose homeostasis has been well investigated in *in vitro*, animal models and some clinical trials [24].

**Figure 1:**
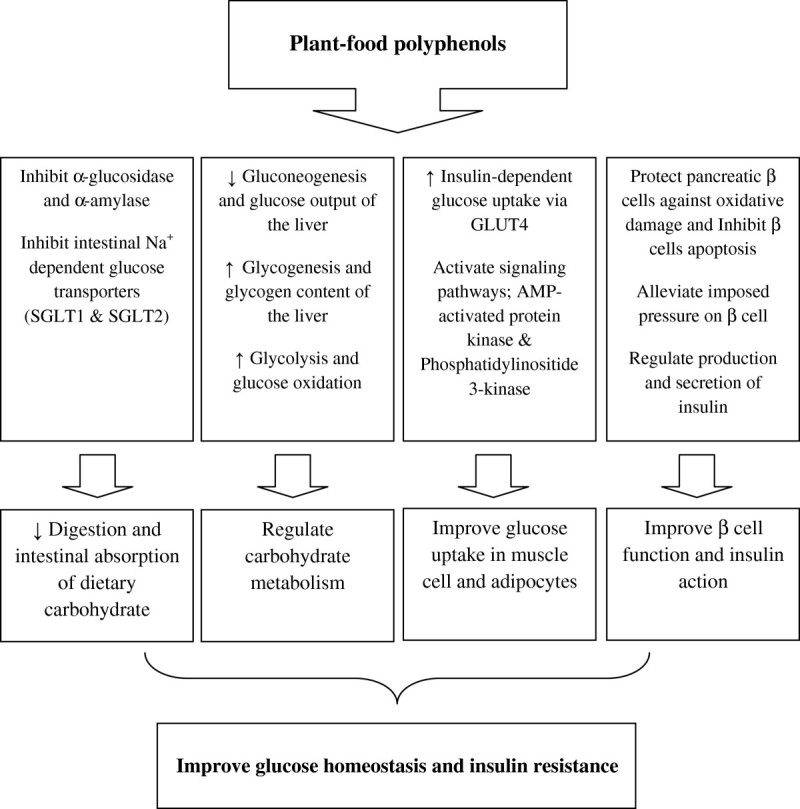
**Beneficial effects of polyphenols on management of blood glucose in diabetes.** The hypoglycemic effects of polyphenols are mainly attributed to reduce intestinal absorption of dietary carbohydrate, modulation of the enzymes involved in glucose metabolism, improvement of β-cell function and insulin action, and stimulation of insulin secretion.

In Figure 1 beneficial effects of polyphenols on management of blood glucose in diabetes are summarized. The hypoglycemic effects of polyphenols are mainly attributed to reduce intestinal absorption of dietary carbohydrate, modulation of the enzymes involved in glucose metabolism, improvement of β-cell function and insulin action, stimulation of insulin secretion, and the antioxidative and anti-inflammatory properties of these components [25–27].

One of the most well known properties of the polyphenols, especially flavonoids, phenolic acids and tannins, on carbohydrate metabolism is inhibition of α-glucosidase and α-amylase, the key enzymes responsible for digestion of dietary carbohydrates to glucose [26, 28]. Some polyphenols, including green tea catechins and epicatechins, chlorogenic acids, Ferulic acids, caffeic and tannic acids, quercetin and naringenin, could interact with absorption of glucose from intestine via inhibition of Na^+^-dependent glucose transporters, SGLT1 and SGLT2 [29, 30]. Some investigations have shown that polyphenolic compounds are also able to regulate postprandial glycemia and inhibit the development of glucose intolerance by a facilitated insulin response and attenuated secretion of glucose-dependent insulinotropic polypeptide (GIP) and glucagon-like polypeptide-1 (GLP-1) [31, 32].

Some polyphenols are able to regulate the key pathways of carbohydrate metabolism and hepatic glucose homeostasis including glycolysis, glycogenesis and gluconeogenesis, usually impaired in diabetes. Ferulic acid, a hydroxycinnamic acid derivate, effectively suppresses blood glucose by elevating glucokinase activity and production of glycogen in the liver and increased plasma insulin levels in diabetic rats [33]. Supplementation of diabetic rats with hesperidin and naringin, two main citrus bioflavonoids, was accompanied with increased hepatic glucokinase activity and glycogen content, attenuated hepatic gluconeogenesis via decrease the activity of glucose-6-phosphatase and phosphoenolpyruvate carboxykinase (PEPCK), and subsequent improvement of glycemic control [34, 35]. Green tea polyphenols, mainly catechins and epicatechins have been shown to attenuate hyperglycemia and hepatic glucose output via downregulation the expression of liver glucokinase and upregulation of PEPCK [36]; in an *in vitro* study, epigallocatechin gallate (EGCG), one of the most abundant catechins in green tea, could activate AMP-activated protein kinase as a required pathway for the inhibition of gluconeogenic enzymes expression [37].

Dietary polyphenols also influence peripheral glucose uptake in both insulin sensitive and non-insulin sensitive tissues; one study showed that phenolic acids stimulated glucose uptake by comparable performance to metformin and thiazolodinedione, main common oral hypoglycemic drugs [38]. The results from the *in vitro* studies showed that some polyphenolic compounds such as quercetin, resveratrol and EGCG improved insulin-dependent glucose uptake in muscle cells and adipocytes by translocation of glucose transporter, GLUT4, to plasma membrane mainly through induction of the AMP-activated protein kinase (AMPK) pathway [39, 40]. AMPK, an important sensor of cellular energy status, has a key role in metabolic control; activation of this pathway is considered as a new treatment for obesity, type 2 diabetes, metabolic syndrome and a main target for anti-diabetic drugs including metformin [41, 42]. Interestingly, effect of polyphenols in activation of AMPK has been reported 50–200 times more than metformin [43]. Some polyphenols also have potential to induce phosphatidylinositide 3-kinase (PI3k) as a key signaling pathway for up-regulation of glucose uptake [44].

Isoflavones, particularly genistein, have amazing effects on pancreatic β-cells; Liu et al reported that anti-diabetic effects of genistein are not associated with stimulation of insulin synthesis, expression of glucose tranporter-2 or glycolytic pathway, genistein acts as a novel agonist of cyclic AMP/protein kinas A signaling, an important physiological amplifier of glucose-induced insulin secretion by the pancreatic β-cells [45, 46]. Furthermore, Fu et al indicated that genistein could induce protein expression of cyclin D1, a major cell-cycle regulator of β-cell growth and subsequently improve islet β-cell proliferation, survival and mass [47].

Hyperglycemia-induced oxidative stress in pancreatic β-cells plays a pivotal role in the development of diabetes [48, 49]. Some of the polyphenolic compounds protect β-cells from hyperglycemia-induced and oxidative-induced damage; oral administration of phenolic-rich chestnut extract in STZ-induced diabetic rats had favorable effects on serum glucose and viability of β-cell through attenuation of oxidative stress, enhancing the natural antioxidant system, and inhibition of lipid peroxidation [50]. Another very known phenolic compound, resveratrol (3,4^′^,5-trihydroxystilbene) found in grapes, wine, grape juice, peanuts, and berries, improve glucose tolerance, attenuate β-cell loss, and reduce oxidative stress in pancreatic islet [51]. Resveratrol also alleviate chronic over stimulation induced-workload and impose pressure on the β-cells, and subsequently delay the degradation of pancreatic islets and progress of type 2 diabetes. This effect appears to be due to the diminished stimulatory effects of hyperglycemia in insulin secretion; some experimental and *in vitro* studies demonstrated that resveratrol has the potential to reduce insulin secretion through induction of metabolic changes in β-cells [51, 52].

Some protective effects of polyphenols on β-cells are related to the ability to modulate key cellular signaling pathways; anthocyanins-rich Chinese bayberry extract showed protective effects for pancreatic β cells against oxidative damage through up-regulation of heme oxygenase-1, modulation of ERK1/2 and PI3K/Akt signaling pathway and inhibition of β cells apoptosis [39].

In summary, results of the studies acknowledge that plant polyphenols favorably affect several aspects of diabetes-induced metabolic disorders and modulate carbohydrate metabolism, glucose homeostasis, insulin secretion and insulin resistance.

### Cardiovascular protective effects of polyphenols with regard to lipid metabolism, blood pressure, blood coagulation and vascular function

Progressive insulin resistance is mainly accompanied with pro-atherogenic cardiovascular risk profiles and consequently atherosclerotic coronary artery disease and other forms of cardiovascular disease are the major causes of mortality in type 2 diabetic patients [53]. Dyslipidemia, undesirable changes in vascular endothelial and smooth muscle cells, lipid peroxidation especially oxidized low-density lipoprotein particles, oxidative damage and increased inflammatory mediators including chemokines and cytokines, hyper-coagulation and platelet activation have been considered as the main metabolic abnormalities in diabetes mellitus leading to cardiovascular disease [54].

There is growing evidence suggesting that dietary intake of polyphenol-rich foods and supplementation with these bioactive components could have protective effects against diabetes-induced cardiovascular pathogenesis; the mechanisms involved in these properties mainly include regulation of lipid metabolism, attenuation of oxidative damage and scavenging of free radicals, improvement of the endothelial function and vascular tone, enhancement the production of vasodilating factors such as nitric oxide, and inhibition the synthesis of vasoconstrictors such as endothelin-1 in endothelial cells [55–57].

One of the most important favorable effects of polyphenols on cardiovascular system in diabetes probably is regulation of lipid and lipoprotein metabolism, and improvement of dyslipidemia. Based on research conducted in this area, polyphenolic compounds are capable of reducing digestion and absorption of dietary lipids. Oligomeric procyanidins, contained in apples had inhibitory effects on pancreatic lipase and triglyceride absorption [58]. Apple procyanidins also induce hypolipidemic effects by decreasing of apolipoprotein B synthesis and secretion, inhibition of cholesterol estrification and intestinal lipoprotein production [59]. The hypolipidemic effects of catechins and proanthcyanidins are related to inhibition of key enzymes in lipid biosynthesis pathways, reduce intestinal lipid absorption. Catechins also interact with proteins involved in cholesterol translocation from the enterocyte brush border (ATP-binding cassette proteins, multidrug resistance P-glycoprotein 1, B type1-scavenger receptors, Niemann Pick C-1 like 1 protein), change their function and effectively reduce cholesterol absorption [60]. Administration of tart cherries as medicinal food rich in anthocyanins was accompanied with decrease in hyperlipidemia, hyperinsulinemia, fatty liver and hepatic steatosis through enhanced hepatic PPARα and some target genes including acyl-coenzyme A oxidase [61].

Endothelial dysfunction, proliferation and migration of smooth muscle cells of the vessels are central events in the pathogenesis of diabetes-induced atherosclerosis. Some cardiovascular protective properties of polyphenols are attributed to modulatory effects on the vascular structure and function. Interestingly, some polyphenols inhibit the expression of major proangiogenic, prothrombotic and proatherosclerotic factors such as monocyte chemoattractant protein-1, vascular endothelial growth factor (VEGF) and matrix metalloproteinase-2 (MMP-2) in smooth muscle cells, by redox-sensitive and redox-insensitive mechanisms [62–64]. Oligomeric proanthcyanidins, found in red apple, cinnamon, cocoa, and grapes have the potential to protect vascular cell against diabetes-induced oxidative stress via increase in activity of superoxide, dismutase inhibition of NADPH oxidase and production of free radicals as well as decrease proliferation of smooth muscle cells [65]. Flanan-3-ols modulate platelet hyperactivity and aggregation, regulate coronary blood flow, reduce endothelial inflammatory cytokines and free radicals, increase production and bioavailability of nitric oxide, and consequently atherosclerosis development [66]. In human trials, consumption of high-polyphenol dark chocolate was accompanied with improvement of endothelial function in individuals with stage 1 hypertension, and attenuation acute transient hyperglycemia-induced endothelial dysfunction and oxidative stress in type 2 diabetic patients [67, 68]. Administration of other polyphenol-rich products such as grape seed extract, cranberry juice, grape and pomegranate juice also had a therapeutic role in decreasing cardiovascular risk factors in patients with type 2 diabetes and metabolic syndrome [69, 70].

Catechins, quercetin and anthocyanins have potent platelet-inhibitory properties and are considered as inhibitors of platelet cell signaling and thrombus formation [71]. As reviewed by Pascul-Tresa, bioactive anthocyanins including delphinidin-3-rotinoside, cyanidin-3-glucoside, cyanidin-3-rutinoside, malvidin-3-glucoside and intestinal metabolites such as dihydroferulic acid and 3- (hydroxyphenyl) propionic acid prevent platelet hyper-activation and aggregation through inhibition of peptides activating thrombin receptor [66].

Undoubtedly, one of the main protective properties of polyphenols in the development of cardiovascular dysfunction in diabetic condition is related to the ability of these bioactive components to prevent lipoproteins oxidation and production of advanced glycation end products. Dietary polyphenolic compounds also protect myocardial tissue against several undesirable changes and diabetic cardiomyopathy. Treatment of diabetic rat models with grape seed proanthcyanidins extracts effectively reduced the receptor of advanced glycation end products (RAGE), nuclear factor-kappaB (NF-kappaB), and transforming growth factor-β (TGF-β) gene expression in myocardial tissue [72].

Human studies have confirmed the benefits of polyphenols on the cardiovascular system; Rizza et al in a randomized cross-over clinical trial shown that oral administration (50 mg/d for 3 week) of hesperidin, a citrus flavonoids, increased flow mediated dilation and reduced circulating inflammatory biomarkers (hs-CRP, serum amyloid A protein, soluble E selectin), decreased adhesion of monocytes, expression of vascular cell adhesion moleculs-1 and generally improved vascular function in patients with metabolic syndrome [73]. Another cross-over trial conducted in postmenopausal type 2 diabetic patients, showed that supplementation with isoflavones from red clover (50 mg/day) after 4 weeks improved endothelial function and decrease systolic and diastolic blood pressure [74].

In summary, results of the studies confirm that polyphenolic compounds attenuate several cardiovascular risk factors in diabetes; dietary polyphenols modulate lipid metabolism and dyslipidemia, improve vascular function, decrease oxidative and inflammatory-induced vascular damage, and regulate blood pressure.

### Antioxidative properties of polyphenols

One of the pathogenic mechanisms that explain the development and progression of micro and macro vascular complications in diabetes is oxidative stress; increased generation of free radicals and an impaired antioxidant defense system in diabetic conditions induces imbalance of the oxidant/antioxidant status [2, 75]. Inhibition of these oxidative processes could prevent the onset and development of long-term diabetic complications [76].

Most polyphenolic compounds and their active metabolites have been known as potent antioxidant phytochemicals due to their unique structure. As reviewed by Dembinska-Kiec, these compounds could scavenge free radicals, quench electronically excited compounds, reduce hydroperoxide formation, and attenuate production of reactive oxygen species (ROS) through modulation of several enzymes involved in the development of ROS including xanthine oxidase, cyclooxygenase, lipoxygenase, microsomal monooxygenase, NADH oxidase and mitochondrial succinoxidase [77]. Some experts believe that polyphenols beyond the direct antioxidant capacities in scavenging of free radicals mainly act by direct interactions with important cellular receptors or key signaling pathways, which may result in modification of the redox status of the cell and may trigger a series of redox-dependent reactions [78].

Plant polyphenols can enhance the endogenous antioxidative system, improve oxidant antioxidant balance, and effectively prevent oxidative damage; green tea catechins are the polyphenolic compounds most studied polyphenolic compounds in this area; these bioactive components decreased lipid peroxidation and increased plasma total antioxidant capacity; they also attenuated of stress-sensitive signaling pathways, prooxidant enzymes, and inducted of antioxidant enzymes including superoxide dismutase, catalase and glutathione peroxidase [79].

In summary, polyphenols effectively attenuate oxidative stress, promote endogenous antioxidant defense system, and modulate oxidant/antioxidant balance.

### Effects of polyphenols on adipose tissue metabolism

Adipocyte dysfunction is strongly associated with the development of insulin resistance, sub-clinical inflammation, β cell impairment and type 2 diabetes [80]. Polyphenolic compounds have wonderful modulatory effects on many aspects of metabolic, endocrine and cellular signaling transduction of adipose tissue. Some polyphenols such as catechins increase β oxidation in adipocytes, down-regulate the enzymes and genes involved in lipogenesis including lipoprotein lipase, fatty acid synthase complex, peroxisome proliferator-activated receptor γ (PPARγ), CCAAT enhancer-binding protein-α, regulatory element-binding protein 1-c, fatty acid binding protein. Some polyphenols, up-regulate lipolysis pathways via induction of hormone sensitive lipase, adipose tissue lipase, increased gene expression of mitochondrial uncoupling protein 2 (UCP-2) and carnitin palmitoyl transferase-1 (CPT-1) in adipocytes [81, 82].

Anthocyanins, one group of phenolic compounds considered as modulators of adipose tissue metabolism, are bioactive components which improve adipocytes dysfunction and adipocytokines secretion in insulin resistance, increase β oxidation and decrease fat accumulation on adipocytes [83]. Cyanidin and cyanidin-3-glucoside have shown several therapeutic effects on adipocyte dysfunction through decrease in plasminogen activator inhibitor-1 and interleukin-6, induction of mitochondrial uncoupling proteins, acetyl CoA oxidase, perlipin and adiponectin gene expression [84].

### Favorable effects of polyphenols in prevention of long-term diabetes complications

One of the most important properties of polyphenols recently identified is its preventive effect against long-term diabetes complications including retinopathy, nephropathy and neuropathy; based on research conducted in this area administration of anthocyanins, flavonoids and other polyphenolic compounds in diabetic conditions may facilitate new approaches for improving the quality of life in diabetic patients.

As reviewed by Ghosh et al, anthocyanins and anthocyanins-rich extracts have the potential to alleviate the developing pathways of some pathologic conditions related to diabetes; anthocyanins facilitate blood flow and prevent diabetes-induced microangiopathy, increase microvascular permeability, decrease leucocytes aggregation in vascular cell wall and improve capillary filtration of albumin [85]. Purple corn extract containing cyanidin 3-glucoside and cyanidin-3-(6″-malonylglucoside) attenuated hyperglycemia-induced mesangial cell proliferation and matrix accumulation, and inhibited over-expression intracellular cell adhesion molecule-1 and monocyte chemoattractant protein-1, as major features of diabetic mesangial fibrosis and glomerulosclerosis [86]. Recently there has been increasing interest in grape seed proanthcyanidin extracts as a natural treatment for some important diabetes complications; proanthcyanidin-rich grape seed extract inhibited the development of retinopathy, nephropathy and neurodegenerative damage in diabetic condition [87, 88].

Flavanols surprisingly have the potential to improve cognitive disorders and cholinergic dysfunction related to diabetes and other secondary consequence of changes in the nervous system induced by hyperglycemia and diabetes oxidative stress; administration of quercetin in diabetic rats improved mental function and memory via inhibition of acetylcholine esterase and attenuation of oxidative damage in nervous system [89].

Green tea catechins including epicatechin, epicatechin-3-gallat, and epigalocatechin gallat decreased the synthesis of thromboxane A2 (TXA2) and increased prostacyclin I2 (PGI2), modulate the impaired balance between these ecosanoids as accelerators of thrombogenesis in the renal tubules, leading consequently improved glomerular filtration and kidney function [90].

## Conclusion

Type 2 diabetes, a clustering of metabolic disorder, is accompanied with other pathogenic conditions including sub-clinical inflammation and oxidative stress that subsequently leads to insulin resistance and long-term diabetes complications. The rising trend in the prevalence of diabetes complications suggests that current medical treatments for the management of diabetes are not sufficient and use of supplementary treatments, including functional foods and their nutraceuticals, could increase the effectiveness of diabetes management. Plant polyphenols including phenolic acids, flavonoids, stilbenes and lignans, based on *in vitro* studies*,* animal models and some clinical trials, have been proposed as effective supplements for diabetes management and prevention of its long-term complications. Further investigations using human clinical studies are needed to confirm the beneficial effects of polyphenolic compounds as supplementary treatments for diabetic patients.

## Authors’ original submitted files for images

Below are the links to the authors’ original submitted files for images. Authors’ original file for figure 1

## Abbreviations

NF-κB: Nuclear factor kappa-B
AP-1: Activator protein-1 DNA binding
ERK: Extracellular signal-regulated protein kinase
PI3: Phosphoinositide 3 kinase/protein kinase B
MAPK: Mitogen-activated protein kinases
Nrf2: Nuclear factor erythroid 2 related factor 2
GIP: Glucose-dependent insulinotropic polypeptide
SGLT: Sodium-dependent glucose transporter
PEPCK: Glucose-6-phosphatase and phosphoenolpyruvate carboxykinase
AMPK: AMP-activated protein kinase
GLUT: Glucose transporter
EGCG: Epigalocatechin gallate
PPARα: Peroxisome proliferator-activated receptor alpha
VEGF: Vascular endothelial growth factor
MMP-2: Matrix metalloproteinase-2
AGEs: Advanced glycation end products
TGF-β: Transforming growth factor-β
ROS: Reactive oxygen species
TXA2: Thromboxane A2
PGI2: Increased prostacyclin I2
UCP-2: Uncoupling protein 2
CPT-1: Carnitin palmitoyl transferase-1.

## References

[1] Santaguida PL, Balion C, Hunt D (2008). Diagnosis, prognosis, and treatment of impaired glucose tolerance and impaired fasting glucose. Evid Rep Technol Assess.

[2] Evans JL, Goldfine ID, Maddux BA, Grodsky GM (2002). Oxidative stress and stress-activated signaling pathways: a unifying hypothesis of type 2 diabetes. Endocr Rev.

[3] Spranger J, Kroke A, Möhlig M, Hoffmann K, Bergmann MM, Ristow M (2003). Inflammatory cytokines and the risk to develop type 2 diabetes: results of the prospective population-based European Prospective Investigation into Cancer and Nutrition (EPIC)-Potsdam Study. Diabetes.

[4] Montonen J, Knekt P, Järvinen R, Reunanen A (2004). Dietary antioxidant intake and risk of type 2 diabetes. Diabetes Care.

[5] Pandey KB, Rizvi SI (2009). Plant polyphenols as dietary antioxidants in human health and disease. Oxid Med Cell Longev.

[6] Bahadoran Z, Golzarand M, Mirmiran P, Saadati N, Azizi F (2013). The association of dietary phytochemical index and cardio-metabolic risk factors in adults: Tehran lipid and glucose study. J Hum Nutr Diet.

[7] Bahadoran Z, Golzarand M, Mirmiran P, Shiva N, Azizi F (2012). Dietary total antioxidant capacity and the occurrence of metabolic syndrome and its components after a 3-year follow-up in adults: Tehran lipid and glucose study. Nutr Metab (Lond).

[8] Mirmiran P, Bahadoran Z, Golzarand M, Shiva N, Azizi F (2012). Association between dietary phytochemical index and 3-year changes in weight, waist circumference and body adiposity index in adults: Tehran lipid and glucose study. Nutr Metab (Lond).

[9] Mirmiran P, Noori N, Zavareh MB, Azizi F (2009). Fruit and vegetable consumption and risk factors for cardiovascular disease. Metabolism.

[10] Perera PK, Li Y (2012). Functional herbal food ingredients used in type 2 diabetes mellitus. Pharmacogn Rev.

[11] Bahadoran Z, Mirmiran P, Hosseinpanah F, Hedayati M, Hosseinpour-Niazi S, Azizi F (2011). Broccoli sprouts reduce oxidative stress in type 2 diabetes: a randomized double-blind clinical trial. Eur J Clin Nutr.

[12] Bahadoran Z, Mirmiran P, Hosseinpanah F, Asghari G, Rajab A, Azizi F (2012). Broccoli sprouts Powder could improve serum triglyceride and oxidized LDL/LDL-cholesterol ratio in type 2 diabetic patients: a randomized double-blind placebo-controlled clinical trial. Diabetes Res Clin Pract.

[13] Bahadoran Z, Tohidi M, Nazeri P, Mehran M, Azizi F, Mirmiran P (2012). Effect of broccoli sprouts on insulin resistance in type 2 diabetic patients: a randomized double-blind clinical trial. Int J Food Sci Nutr.

[14] Beckman CH (2000). Phenolic-storing cells: keys to programmed cell death and periderm formation in wilt disease resistance and in general defence responses in plants?. Physiol Mol Plant Pathol.

[15] Pietta P, Minoggio M, Bramati L (2003). Plant polyphenols: structure, occurrence and bioactivity. Stud Nat Pro Chem.

[16] Manach C, Scalbert A, Morand C, Rémésy C, Jiménez L (2004). Polyphenols: food sources and bioavailability. Am J Clin Nutr.

[17] Adlercreutz H (2007). Lignans and human health. Crit Rev Clin Lab Sci.

[18] Chun OK, Chung SJ, Song WO (2007). Estimated dietary flavonoid intake and major food sources of U.S. adults. J Nutr.

[19] Ovaskainen ML, Törrönen R, Koponen JM, Sinkko H, Hellström J, Reinivuo H (2008). Dietary intake and major food sources of polyphenols in Finnish adults. J Nutr.

[20] Scalbert A, Williamson G (2000). Dietary intake and bioavailability of polyphenols. J Nutr.

[21] Scalbert A, Morand C, Manach C, Rémésy C (2002). Absorption and metabolism of polyphenols in the gut and impact on health. Biomed Pharmacother.

[22] Han X, Loa T (2007). Dietary polyphenols and their biological significance. Int J Mol Sci.

[23] Dinneen S, Gerich J, Rizza R (1992). Carbohydrate metabolism in non-insulin-dependent diabetes mellitus. N Engl J Med.

[24] Hanhineva K, Törrönen R, Bondia-Pons I, Pekkinen J, Kolehmainen M, Mykkänen H (2010). Impact of dietary polyphenols on carbohydrate metabolism. Int J Mol Sci.

[25] Iwai K, Kim MY, Onodera A, Matsue H (2006). Alpha-glucosidase inhibitory and antihyperglycemic effects of polyphenols in the fruit of Viburnum dilatatum Thunb. Agric Food Chem.

[26] Iwai K (2008). Antidiabetic and antioxidant effects of polyphenols in brown alga Ecklonia stolonifera in genetically diabetic KK-A(y) mice. Plant Foods Hum Nutr.

[27] Cabrera C, Artacho R, Giménez R (2006). Beneficial effects of green tea-a review. J Am Coll Nutr.

[28] Tadera K, Minami Y, Takamatsu K, Matsuoka T (2006). Inhibition of alpha-glucosidase and alpha-amylase by flavonoids. J Nutr Sci Vitaminol (Tokyo).

[29] Kobayashi Y, Suzuki M, Satsu H, Arai S, Hara Y, Suzuki K (2000). Green tea polyphenols inhibit the sodium-dependent glucose transporter of intestinal epithelial cells by a competitive mechanism. J Agric Food Chem.

[30] Johnston K, Sharp P, Clifford M, Morgan L (2005). Dietary polyphenols decrease glucose uptake by human intestinal Caco-2 cells. FEBS Lett.

[31] Johnston KL, Clifford MN, Morgan LM (2003). Coffee acutely modifies gastrointestinal hormone secretion and glucose tolerance in humans: glycemic effects of chlorogenic acid and caffeine. Am J Clin Nutr.

[32] Dao TM, Waget A, Klopp P, Serino M, Vachoux C, Pechere L (2011). Resveratrol increases glucose induced GLP-1 secretion in mice: a mechanism which contributes to the glycemic control. PLoS One.

[33] Jung EH, Kim SR, Hwang IK, Ha TY (2007). Hypoglycemic effects of a phenolic acid fraction of rice bran and ferulic acid in C57BL/KsJ-db/db mice. J Agric Food Chem.

[34] Jung UJ, Lee MK, Jeong KS, Choi MS (2004). The hypoglycemic effects of hesperidin and naringin are partly mediated by hepatic glucose-regulating enzymes in C57BL/KsJ-db/db mice. J Nutr.

[35] Jung UJ, Lee MK, Park YB, Kang MA, Choi MS (2006). Effect of citrus flavonoids on lipid metabolism and glucose-regulating enzyme mRNA levels in type-2 diabetic mice. Int J Biochem Cell Biol.

[36] Waltner-Law ME, Wang XL, Law BK, Hall RK, Nawano M, Granner DK (2002). Epigallocatechin gallate, a constituent of green tea, represses hepatic glucose production. J Biol Chem.

[37] Collins QF, Liu HY, Pi J, Liu Z, Quon MJ, Cao W (2007). Epigallocatechin-3-gallate (EGCG), a green tea polyphenol, suppresses hepatic gluconeogenesis through 5′-AMP-activated protein kinase. J Biol Chem.

[38] Prabhakar PK, Doble M (2009). Synergistic effect of phytochemicals in combination with hypoglycemic drugs on glucose uptake in myotubes. Phytomedicine.

[39] Zhang B, Kang M, Xie Q, Xu B, Sun C, Chen K, Wu Y (2011). Anthocyanins from Chinese bayberry extract protect β cells from oxidative stress-mediated injury via HO-1 upregulation. J Agric Food Chem.

[40] Park CE, Kim MJ, Lee JH, Min BI, Bae H, Choe W (2007). Resveratrol stimulates glucose transport in C2C12 myotubes by activating AMP-activated protein kinase. Mol Med.

[41] Towler MC, Hardie DG (2007). AMP-activated protein kinase in metabolic control and insulin signaling. Circ Res.

[42] Zhou G, Myers R, Li Y, Chen Y, Shen X, Fenyk-Melody J (2001). Role of AMP-activated protein kinase in mechanism of metformin action. J Clin Invest.

[43] Zang M, Xu S, Maitland-Toolan KA, Zuccollo A, Hou X, Jiang B (2006). Polyphenols stimulate AMP-activated protein kinase, lower lipids, and inhibit accelerated atherosclerosis in diabetic LDL receptor-deficient mice. Diabetes.

[44] Kumar R, Balaji S, Uma TS, Sehgal PK (2009). Fruit extracts of Momordica charantia potentiate glucose uptake and up-regulate Glut-4, PPAR gamma and PI3K. J Ethnopharmacol.

[45] Fu Z, Liu D (2009). Long-term exposure to genistein improves insulin secretory function of pancreatic beta-cells. Eur J Pharmacol.

[46] Liu D, Zhen W, Yang Z, Carter JD, Si H, Reynolds KA (2006). Genistein acutely stimulates insulin secretion in pancreatic beta-cells through a cAMP-dependent protein kinase pathway. Diabetes.

[47] Fu Z, Zhang W, Zhen W, Lum H, Nadler J, Bassaganya-Riera J (2010). Genistein induces pancreatic beta-cell proliferation through activation of multiple signaling pathways and prevents insulin-deficient diabetes in mice. Endocrinology.

[48] Kajimoto Y, Kaneto H (2004). Role of oxidative stress in pancreatic beta-cell dysfunction. Ann NY Acad Sci.

[49] Drews G, Krippeit-Drews P, Düfer M (2010). Oxidative stress and beta-cell dysfunction. Pflugers Arch.

[50] Yin P, Zhao S, Chen S, Liu J, Shi L, Wang X, Liu Y, Ma C (2011). Hypoglycemic and hypolipidemic effects of polyphenols from burs of Castanea mollissima Blume. Molecules.

[51] Szkudelski T, Szkudelska K (2011). Anti-diabetic effects of resveratrol. Ann NY Acad Sci.

[52] Szkudelski T (2006). Resveratrol inhibits insulin secretion from rat pancreatic islets. Eur J Pharmacol.

[53] Kalofoutis C, Piperi C, Kalofoutis A, Harris F, Phoenix D, Singh J (2007). Type II diabetes mellitus and cardiovascular risk factors: Current therapeutic approaches. Exp Clin Cardiol.

[54] Thomas JE, Foody JM (2007). The pathophysiology of cardiovascular disease in diabetes mellitus and the future of therapy. J Cardiometab Syndr.

[55] Lecour S, Lamont KT (2011). Natural polyphenols and cardioprotection. Mini Rev Med Chem.

[56] Schini-Kerth VB, Auger C, Etienne-Selloum N, Chataigneau T (2010). Polyphenol-induced endothelium-dependent relaxations role of NO and EDHF. Adv Pharmacol.

[57] Stoclet JC, Chataigneau T, Ndiaye M, Oak MH, El Bedoui J, Chataigneau M (2004). Vascular protection by dietary polyphenols. Eur J Pharmacol.

[58] Sugiyama H, Akazome Y, Shoji T, Yamaguchi A, Yasue M, Kanda T (2007). Oligomeric procyanidins in apple polyphenol are main active components for inhibition of pancreatic lipase and triglyceride absorption. J Agric Food Chem.

[59] Vidal R, Hernandez-Vallejo S, Pauquai T, Texier O, Rousset M, Chambaz J (2005). Apple procyanidins decrease cholesterol esterification and lipoprotein secretion in Caco-2/TC7 enterocytes. J Lipid Res.

[60] Koo SI, Noh SK (2007). Green tea as inhibitor of the intestinal absorption of lipids: potential mechanism for its lipid-lowering effect. J Nutr Biochem.

[61] Seymour EM, Singer AA, Kirakosyan A, Urcuyo-Llanes DE, Kaufman PB, Bolling SF (2008). Altered hyperlipidemia, hepatic steatosis, and hepatic peroxisome proliferator-activated receptors in rats with intake of tart cherry. J Med Food.

[62] Oak MH, Chataigneau M, Keravis T, Chataigneau T, Beretz A, Andriantsitohaina R (2003). Red wine polyphenolic compounds inhibit vascular endothelial growth factor expression in vascular smooth muscle cells by preventing the activation of the p38 mitogen-activated protein kinase pathway. Arterioscler Thromb Vasc Biol.

[63] Oak MH, El Bedoui J, Schini-Kerth VB (2005). Antiangiogenic properties of natural polyphenols from red wine and green tea. J Nutr Biochem.

[64] Ndiaye M, Chataigneau T, Chataigneau M, Schini-Kerth VB (2004). Red wine polyphenols induce EDHF-mediated relaxations in porcine coronary arteries through the redox-sensitive activation of the PI3-kinase/Akt pathway. Br J Pharmacol.

[65] Wang L, Zhu LH, Jiang H, Tang QZ, Yan L, Wang D (2010). Grape seed proanthocyanidins attenuate vascular smooth muscle cell proliferation via blocking phosphatidylinositol 3-kinase-dependent signaling pathways. J Cell Physiol.

[66] de Pascual-Teresa S, Moreno DA, García-Viguera C (2010). Flavanols and anthocyanins in cardiovascular health: a review of current evidence. Int J Mol Sci.

[67] Nogueira Lde P, Knibel MP, Torres MR, Nogueira Neto JF, Sanjuliani AF (2012). Consumption of high-polyphenol dark chocolate improves endothelial function in individuals with stage 1 hypertension and excess body weight. Int J Hypertens.

[68] Mellor DD, Madden LA, Smith KA, Kilpatrick ES, Atkin SL (2012). High-polyphenol chocolate reduces endothelial dysfunction and oxidative stress during acute transient hyperglycaemia in Type 2 diabetes: a pilot randomized controlled trial. Diabet Med.

[69] Kar P, Laight D, Rooprai HK, Shaw KM, Cummings M (2009). Effects of grape seed extract in Type 2 diabetic subjects at high cardiovascular risk: a double blind randomized placebo controlled trial examining metabolic markers, vascular tone, inflammation, oxidative stress and insulin sensitivity. Diabet Med.

[70] Shidfar F, Heydari I, Hajimiresmaiel SJ, Hosseini S, Shidfar S, Amiri F (2012). The effects of cranberry juice on serum glucose, apoB, apoA-I, Lp(a), and Paraoxonase-1 activity in type 2 diabetic male patients. J Res Med Sci.

[71] Hubbard GP, Wolffram S, Lovegrove JA, Gibbins JM (2004). Ingestion of quercetin inhibits platelet aggregation and essential components of the collagen-stimulated platelet activation pathway in humans. J Thromb Haemost.

[72] Cheng M, Gao HQ, Xu L, Li BY, Zhang H, Li XH (2007). Cardioprotective effects of grape seed proanthocyanidins extracts in streptozocin induced diabetic rats. J Cardiovasc Pharmacol.

[73] Rizza S, Muniyappa R, Iantorno M, Kim JA, Chen H, Pullikotil P (2011). Citrus polyphenol hesperidin stimulates production of nitric oxide in endothelial cells while improving endothelial function and reducing inflammatory markers in patients with metabolic syndrome. J Clin Endocrinol Metab.

[74] Howes JB, Tran D, Brillante D, Howes LG (2003). Effects of dietary supplementation with isoflavones from red clover on ambulatory blood pressure and endothelial function in postmenopausal type 2 diabetes. Diabetes Obes Metab.

[75] Goycheva V, Gadjeva BP (2006). Oxidative stress and its complications in diabetes mellitus. Trakia J of Sci.

[76] Pérez-Matute P, Zulet MA, Martínez JA (2009). Reactive species and diabetes: counteracting oxidative stress to improve health. Curr Opin Pharmacol.

[77] Dembinska-Kiec A, Mykkänen O, Kiec-Wilk B, Mykkänen H (2008). Antioxidant phytochemicals against type 2 diabetes. Br J Nutr.

[78] Scalbert A, Johnson IT, Saltmarsh M (2005). Polyphenols: antioxidants and beyond. Am J Clin Nutr.

[79] Crespy V, Williamson G (2004). A review of the health effects of green tea catechins in in vivo animal models. J Nutr.

[80] Guilherme A, Virbasius JV, Puri V, Czech MP (2008). Adipocyte dysfunctions linking obesity to insulin resistance and type 2 diabetes. Nat Rev Mol Cell Biol.

[81] Nakazato K, Song H, Waga T (2006). Effects of dietary apple polyphenol on adipose tissues weights in Wistar rats. Exp Anim.

[82] Osada K, Suzuki T, Kawakami Y, Senda M, Kasai A, Sami M (2006). Dose-dependent hypocholesterolemic actions of dietary apple polyphenol in rats fed cholesterol. Lipids.

[83] Tsuda T, Horio F, Uchida K, Aoki H, Osawa T (2003). Dietary cyanidin 3-O-beta-D-glucoside-rich purple corn color prevents obesity and ameliorates hyperglycemia in mice. J Nutr.

[84] Tsuda T, Ueno Y, Yoshikawa T, Kojo H, Osawa T (2006). Microarray profiling of gene expression in human adipocytes in response to anthocyanins. Biochem Pharmacol.

[85] Ghosh D, Konishi T (2007). Anthocyanins and anthocyanin-rich extracts: role in diabetes and eye function. Asia Pac J Clin Nutr.

[86] Li J, Lim SS, Lee JY, Kim JK, Kang SW, Kim JL (2012). Purple corn anthocyanins dampened high-glucose-induced mesangial fibrosis and inflammation: possible renoprotective role in diabetic nephropathy. J Nutr Biochem.

[87] Li BY, Cheng M, Gao HQ, Ma YB, Xu L, Li XH (2008). Back-regulation of six oxidative stress proteins with grape seed proanthocyanidin extracts in rat diabetic nephropathy. J Cell Biochem.

[88] Cui XP, Li BY, Gao HQ, Wei N, Wang WL, Lu M (2008). Effects of grape seed proanthocyanidin extracts on peripheral nerves in streptozocin-induced diabetic rats. J Nutr Sci Vitaminol (Tokyo).

[89] Bhutada P, Mundhada Y, Bansod K, Bhutada C, Tawari S, Dixit P (2010). Ameliorative effect of quercetin on memory dysfunction in streptozotocin-induced diabetic rats. Neurobiol Learn Mem.

[90] Rhee SJ, Choi JH, Park MR (2002). Green tea catechin improves microsomal phospholipase A2 activity and the arachidonic acid cascade system in the kidney of diabetic rats. Asia Pac J Clin Nutr.

