# Supplementary material for: Dietary polyphenols as potential nutraceuticals in management of diabetes: a review
Source: J Diabetes Metab Disord. 2013 Aug 13;12:43. doi: 10.1186/2251-6581-12-43 (PMC7968452; doi:10.1186/2251-6581-12-43)
Supplement: Supplementary file 1 — Authors’ original file for figure 1 [file 40200_2013_73_MOESM1_ESM.pdf]

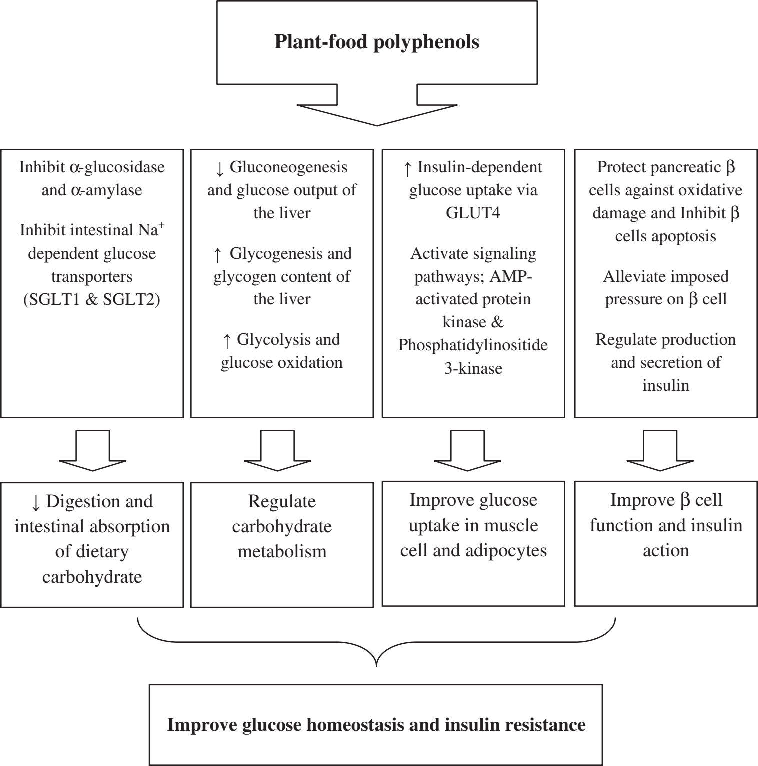
Plant-food polyphenols
Inhibit α-glucosidase and α-amylase
Inhibit intestinal Na^+ dependent glucose transporters (SGLT1 & SGLT2)
↓ Gluconeogenesis and glucose output of the liver
↑ Glycogenesis and glycogen content of the liver
↑ Glycolysis and glucose oxidation
↑ Insulin-dependent glucose uptake via GLUT4
Activate signaling pathways; AMP-activated protein kinase & Phosphatidylinositide 3-kinase
Protect pancreatic β cells against oxidative damage and Inhibit β cells apoptosis
Alleviate imposed pressure on β cell
Regulate production and secretion of insulin
↓ Digestion and intestinal absorption of dietary carbohydrate
Regulate carbohydrate metabolism
Improve glucose uptake in muscle cell and adipocytes
Improve β cell function and insulin action
Improve glucose homeostasis and insulin resistance
